# Non-Scar Fixation Technique for Zygomaticomaxillary Complex Tripod Fracture Using Upper Lid Transconjunctival Incision

**DOI:** 10.1055/a-2875-5296

**Published:** 2026-05-29

**Authors:** Han Gyu Cha, Seo Koo Lee, Joon Suk Bae, Seung Min Nam, Eun Soo Park

**Affiliations:** College of Medicine, Department of Plastic and Reconstructive Surgery 26730 Soonchunhyang University Bucheon Hospital, Soonchunhyang UniversityBucheonKorea

**Keywords:** fracture-skull, zygomatic fractures, orbital fractures, fracture fixation, surgical procedures

## Abstract

**Background:**

Surgical approaches for zygomaticomaxillary complex (ZMC) fractures have evolved toward minimizing cutaneous incisions while maintaining adequate exposure. Conventional approaches for frontozygomatic fixation, such as lateral brow or transconjunctival incisions with lateral extension, may result in visible scarring or periocular complications. Building on previous transconjunctival techniques, we applied an independent upper eyelid transconjunctival incision as a non-cutaneous approach for frontozygomatic fixation in selected ZMC tripod fractures.

**Methods:**

This retrospective comparative study included 27 patients with unilateral ZMC tripod fractures, of whom 12 underwent non-scar fixation using an upper eyelid transconjunctival approach, and 15 underwent fixation through preexisting periorbital traumatic lacerations. The surgical strategy consisted of initial reduction and fixation via an intraoral approach, followed by selective additional fixation of the frontozygomatic region when residual step deformity or instability was present. Preoperative and 6-month postoperative computed tomography and three-dimensional photogrammetry were used to evaluate bony and soft tissue intermalar height differences.

**Results:**

Preoperative intermalar height differences were comparable between groups. At 6 months postoperatively, both groups showed marked improvement, with bony differences of −0.2 ± 1.2 mm and −0.4 ± 0.4 mm, and soft tissue differences of 0.0 ± 0.6 mm and −0.2 ± 0.7 mm in the non-scar and control groups, respectively, with no clinically meaningful between-group differences. No clinically significant approach-related ophthalmic complications were observed.

**Conclusions:**

The upper eyelid transconjunctival approach is a reliable non-cutaneous option for frontozygomatic fixation in selected ZMC tripod fractures, providing adequate exposure and stable fixation without visible scarring.

## Introduction

The zygoma articulates with four bones through the zygomaticomaxillary, frontozygomatic, zygomaticotemporal, and zygomaticosphenoidal sutures. Because these articulations form part of the midface buttress system, the zygomaticomaxillary complex (ZMC) is an important functional and aesthetic landmark of the midface. Owing to this complex anatomy, ZMC fractures typically involve the zygomaticomaxillary buttress, zygomatic arch, orbital rim, and zygomaticofrontal buttress.

Open reduction and internal fixation (ORIF) has been the standard treatment for ZMC fractures, and one-, two-, or three-point fixation has been used depending on fracture stability. 1 2 In tripod fractures, two or three fixation points—including the zygomaticomaxillary buttress and the frontozygomatic suture (lateral orbital rim)—are commonly stabilized because these structures serve as important vertical buttresses of the midface. 3 Incisions for frontozygomatic suture fixation commonly include the lateral brow incision, transconjunctival approach with lateral canthotomy, and upper eyelid blepharoplasty incision, all of which may be associated with visible scarring or aesthetic concerns. Langsdon et al described an upper lid transconjunctival approach for exposure of the lateral orbital rim in conjunction with lower eyelid transconjunctival access to the infraorbital rim. 4 Building on this concept, we used an upper eyelid transconjunctival incision as a direct approach to the frontozygomatic region in selected ZMC tripod fractures, regardless of the need for infraorbital rim exposure.

## Methods

This retrospective study was approved by our institutional review board (number 2023-06-012-001). Between October 2014 and December 2022, 27 patients with unilateral ZMC tripod fractures and displacement of the zygomaticofrontal buttress were included. The exclusion criteria were as follows: (1) Comminuted fractures, (2) bilateral ZMC fractures, (3) accompanying mandibular fractures, and (4) patients younger than 18 years. The control group consisted of patients who had preoperative external wounds in the periorbital area and underwent surgery through these wounds. Among the 27 patients, 15 were assigned to the control group with external periorbital wounds, while 12 patients had no external wounds.

### Clinical Evaluation

All patients underwent preoperative three-dimensional (3D) computed tomography (CT) and photogrammetric analysis using a 3D camera (Morpheus3D, Morpheus3D Co., Ltd., South Korea) to evaluate bilateral differences in bony and soft tissue intermalar heights, as well as zygomaticosphenoidal suture (ZSS) alignment.

To measure the difference in bony intermalar height, the axial slice of the 3D-CT scan demonstrating the most prominent portion of the zygoma body was selected, and a reference line parallel to both pyriform apertures was used. To measure the soft tissue intermalar height, an arbitrary reference line parallel to the intercanthal line was drawn through the most prominent malar point on the unfractured side. 5 The distance from the reference line to the most prominent malar region of the fractured side was defined as the difference in soft tissue intermalar heights. Postoperative bony and soft tissue intermalar height assessments were conducted 6 months after surgery.

To evaluate the zygomaticosphenoidal (ZS) alignment, the ZS angle was measured on axial CT images. The ZS angle was defined as the angle between the midsagittal plane and a tangential line along the zygomaticosphenoid suture adjacent to the sphenoid trigone. 6 Measurements were performed on axial CT slices containing the equator of the eyeball, where the zygomaticosphenoid suture and the sphenoid trigone were simultaneously identifiable. The ZS angle was measured bilaterally on the fractured and unfractured sides, and the absolute difference between the two sides was calculated to assess postoperative alignment.

Complications were categorized as major, minor, and approach-related ophthalmic complications. Major complications were defined as those requiring additional surgical or medical intervention or resulting in persistent functional impairment, including wound infection, plate exposure, foreign body reaction, facial asymmetry, and trismus. Minor complications were defined as transient postoperative findings that were resolved spontaneously without intervention, such as chemosis and subconjunctival hemorrhage. Approach-related ophthalmic complications were specifically evaluated due to the use of the transconjunctival approach and included ptosis, eyelid closure dysfunction, tear dysfunction, lacrimal gland-related injury, conjunctival scarring, and lateral canthal or soft tissue asymmetry. These complications were assessed based on postoperative clinical examination and medical record review.

### Surgical Technique

**Supplementary Video 1** Upper eyelid transconjunctival approach for frontozygomatic suture fixation in a left zygomaticomaxillary complex tripod fracture. A conjunctival incision was made superior to the lateral border of the upper tarsal plate, followed by periosteal incision and subperiosteal dissection to expose the zygomaticofrontal suture. Rigid fixation was achieved using a titanium microplate. The periosteum and conjunctiva were closed in a layered fashion.


The overall surgical strategy was based on selective additional exposure after initial reduction through an intraoral approach. In all patients, the procedure was started with a Keen's approach, through which the fractured zygoma was reduced using an elevator. After initial reduction, the infraorbital rim and zygomaticofrontal suture were palpated to assess residual step deformity. Fixation was then performed at the zygomaticomaxillary buttress, followed by repeated assessment of the infraorbital rim and zygomaticofrontal region. If no residual step deformity was palpable and reduction was considered stable, no further exposure was performed. However, if additional reduction was required or if the reduction remained unstable at the infraorbital rim or zygomaticofrontal suture even after buttress fixation, these areas were selectively opened for further reduction and fixation.

For fixation of the lateral orbital rim, an upper eyelid transconjunctival approach was used. After insertion of a corneal protector, the fracture site at the zygomaticofrontal buttress was identified by palpation through the upper eyelid conjunctiva. Silk tagging sutures were placed at the lateral aspects of the upper and lower tarsal plates and used to retract the eyelids superiorly and inferiorly, thereby exposing the lateral conjunctival area. The conjunctiva was retracted laterally using a Crile retractor, while a malleable retractor was positioned along the inner aspect of the lateral orbital rim to provide countertraction and protect the globe. These opposing retractors created adequate tension to stretch the conjunctiva and allow it to be closely apposed to the lateral orbital rim. A conjunctival incision of approximately 1.5 cm was made in the lateral upper eyelid, just superior to the lateral border of the upper tarsal plate. After reaching the periosteum, it was incised, and subperiosteal dissection was performed using a periosteal elevator to expose the lateral orbital rim. Care was taken to minimize injury to the surrounding soft tissues.

**Fig. 1:**
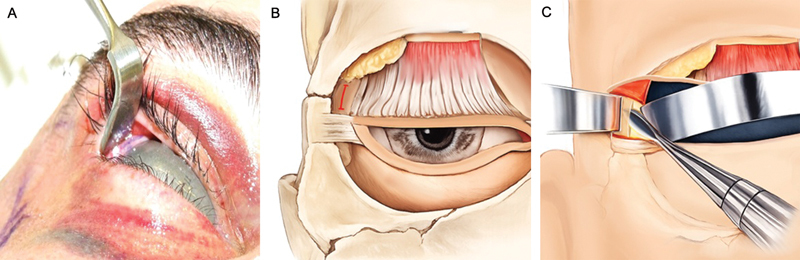
Intraoperative clinical photograph and schematic illustrations demonstrating the upper eyelid transconjunctival approach for frontozygomatic suture fixation in a 42-year-old male patient with a right ZMC tripod fracture. ( **A** ) The incision design was drawn on the conjunctiva of the upper lateral eyelid, superior to Whitnall's tubercle. ( **B** ) Schematic illustration showing the location of the upper eyelid transconjunctival incision (red line). The incision is placed within a safe anatomical corridor that allows access to the frontozygomatic suture while avoiding injury to the lateral canthal tendon, lacrimal gland, and levator aponeurosis. ( **C** ) Schematic illustration of the surgical view after conjunctival incision, demonstrating entry into the preseptal plane and subperiosteal dissection of the lateral orbital rim using an elevator. Both schematic illustrations were created by the authors. ZMC,

The fracture line at the zygomaticofrontal suture was identified under direct visualization, and additional reduction was performed if residual displacement was present. Rigid fixation was then achieved using a straight 1.5-mm 3- or 4-hole titanium microplate, with placement of at least two screws on each side of the fracture line whenever feasible. Finally, the periosteum and conjunctiva were closed in a layered fashion, and the gingivobuccal incision site was repaired ( Fig. 1 and Supplementary Video S1 [available in the online version only]).

### Statistical Analysis

Statistical analyses were performed using IBM SPSS Statistics version 20.0 (IBM Corp., Armonk, NY, United States). The independent-samples *t* -test was used to compare differences in bony and soft tissue intermalar heights between the fractured and unfractured sides, and statistical significance was defined as *p* < 0.05.

## Results

The mean ages of patients in the non-scar fixation and control groups were 33.8 and 43.7 years, respectively. Isolated ZMC fractures were observed in 6 of 12 patients in the non-scar fixation group and 9 of 15 patients in the control group ( Table 1 ).

**Table 1:** Demographics

	Non-scar technique group ( *N* = 12)	Control group ( *N* = 15)	*p* -Value
Age (years)	33.8	43.7	0.02
Sex			
Male	9	12	–
Female	3	3	
Associated fracture			
None	6	9	–
Nasal bone	5	4	
Orbital wall	5	5	
Frontal sinus	0	1	

Preoperative bony intermalar height differences between the fractured and unfractured sides were −2.4 ± 1.2 mm in the non-scar fixation group and −2.8 ± 1.3 mm in the control group. Corresponding soft tissue intermalar height differences were −1.9 ± 1.1 mm and −2.3 ± 1.2 mm, respectively. There was no significant difference in preoperative measurements between the two groups.

**Fig. 2:**
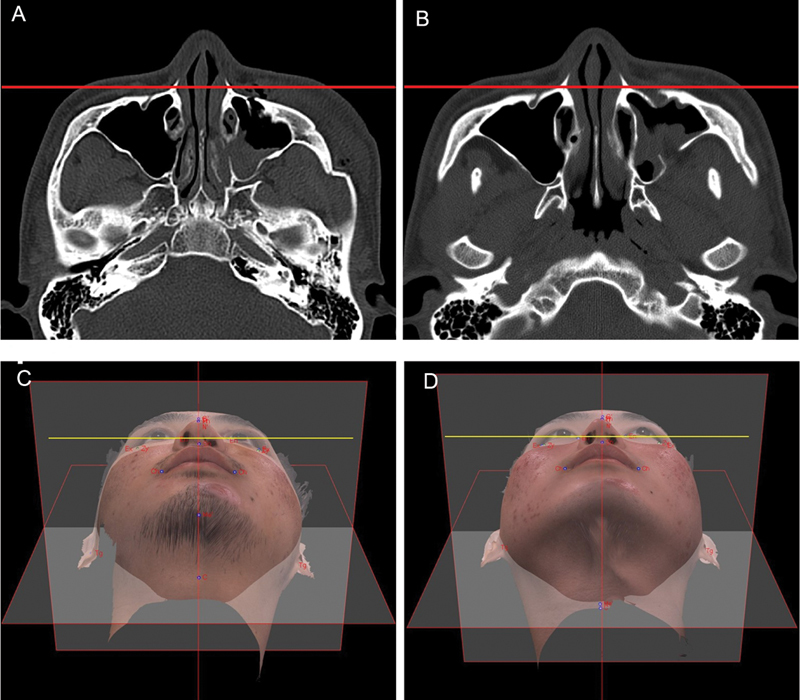
Preoperative and postoperative evaluations of bony and soft tissue intermalar heights between the fractured and unfractured sides in a 21-year-old male patient. ( **A, B** ) The differences in bony intermalar heights were 5 mm preoperatively and 1 mm at 6 months postoperatively. ( **C, D** ) The differences in soft tissue intermalar heights were 2 mm preoperatively and 0 mm at 6 months postoperatively.

**Fig. 3:**
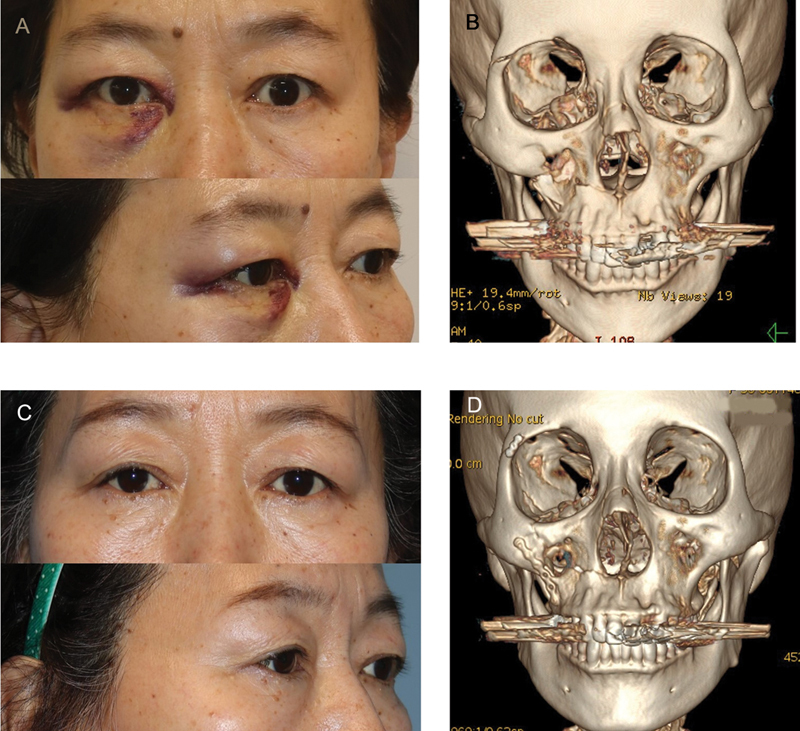
Preoperative and postoperative clinical results of non-scar fixation in ZMC tripod fractures in a 51-year-old female patient. ( **A** ) Preoperative clinical photograph and ( **B** ) three-dimensional computed tomography (3D-CT) scan demonstrating a ZMC tripod fracture combined with a nasal bone fracture. ( **C** ) Postoperative clinical photograph and ( **D** ) 3D-CT scan showing a well-reduced fracture without any scars. ZMC,

At 6 months postoperatively, the mean bony intermalar height difference was −0.2 ± 1.2 mm in the non-scar fixation group and −0.4 ± 0.4 mm in the control group. The corresponding soft tissue intermalar height differences were 0.0 ± 0.6 mm in the non-scar fixation group and −0.2 ± 0.7 mm in the control group ( Figs. 2 and 3 ). Both groups showed postoperative improvement in bony and soft tissue intermalar height differences.

Preoperatively, the ZSS angle difference between the fractured and unfractured sides was 2.79 ± 3.57 degrees in the non-scar fixation group and 2.17 ± 1.81 degrees in the control group. At 6 months postoperatively, the ZSS angle difference improved to 1.54 ± 1.10 degrees and 0.87 ± 0.51 degrees in the non-scar fixation and control groups, respectively, indicating comparable restoration of ZSS alignment between the two groups ( *p* > 0.05; Table 2 ).

**Table 2:** Differences in bony and soft tissue intermalar heights and zygomaticosphenoidal suture alignment between the fractured and unfractured sides

	Non-scar technique group	Control group	*p* -Value
Bony intermalar height (mm)			
Preoperative	−2.4 ± 1.2	−2.8 ± 1.3	0.44
POD 6 months	−0.2 ± 0.3	−0.4 ± 0.4	0.30
Soft tissue intermalar height (mm)			
Preoperative	−1.9 ± 1.1	−2.3 ± 1.2	0.43
POD 6 months	0.0 ± 0.6	−0.2 ± 0.7	0.55
ZSS alignment			
Preoperative	2.79 ± 3.57	2.17 ± 1.81	0.77
POD 6 months	1.54 ± 1.10	0.87 ± 0.51	0.26

Abbreviations: POD, postoperative day; ZSS, zygomaticosphenoidal suture.

All variables are expressed as means ± standard deviation.

No major complications, including wound infection, plate exposure, foreign body reaction, facial asymmetry, or trismus, were observed during the 6-month postoperative follow-up period. Minor complications were limited to transient conjunctival findings. Mild postoperative chemosis occurred in three patients in the non-scar fixation group, all of whom resolved spontaneously within 5 days after surgery. Subconjunctival hemorrhage was observed in two patients in the non-scar fixation group and resolved spontaneously within 5 days without intervention. With respect to approach-related ophthalmic complications, no clinically significant ptosis, impairment of eyelid closure, tear dysfunction, lacrimal gland-related abnormality, conjunctival scarring, or lateral canthal and soft tissue asymmetry was observed during follow-up. There was no significant difference in approach-related ophthalmic complications between the two groups.

## Discussion

ORIF has been used as the standard method for ZMC fractures, and the choice of the incision type is directly related to the fixation point of the fractured site. The gingivobuccal sulcus incision is most widely used because it is relatively simple and useful for approaching the zygomaticomaxillary buttress without scarring. Temporal, bicoronal, subciliary, transconjunctival (with or without lateral canthotomy), and lateral brow incisions are also used to reduce and fix different types of ZMC fractures. 7 8 9

The number of fixation points remains debatable, particularly in tripod fractures. In cases of medium- or high-energy fractures, including tripod fractures classified by Manson et al, two or more fixation points are usually needed to obtain sufficient rigidity and stability. 10 However, some authors still insist that one-point fixation is sufficient for tripod fractures. Fujioka et al suggested that one-plate fixation of the zygomaticomaxillary buttress provides sufficient rigidity by achieving three-point alignment, except in comminuted cases. 11 Other authors have reported that one-point fixation of the zygomaticofrontal buttress provides acceptable stability for ZMC fractures. 12 13 Both techniques have some limitations. In most zygomatic fractures, there is only a small space for fixation at the maxillary edge of the zygomaticomaxillary buttress region, and inserting two or more screws for fixation at this region is difficult. 14 Rotation of the fractured segment may occur when fixation is performed only at the inferior limb of the ZMC. Fixation of only the zygomaticofrontal suture line failed to achieve a stable position of the malar prominence. Although anatomical constraints occasionally limited ideal screw placement in our cases, stable fixation was achieved without postoperative displacement. We acknowledge these constraints as a technical limitation of the procedure.

Accordingly, support from both vertical buttresses is required to maintain stable midfacial height in tripod fractures. Two-point fixation at the frontozygomatic suture and the zygomaticomaxillary buttress has, therefore, been advocated as an effective method. However, traditional incisions for the fixation of the zygomaticofrontal area have certain limitations. A lateral eyebrow incision may result in visible scarring and postoperative swelling due to manipulation of the orbicularis oculi muscle, which can negatively affect patient satisfaction. For Asians, who have a skin texture with a higher tendency of scarring than Caucasians, scarring is one of the most important factors when choosing the surgical technique. Several authors have introduced upper lateral blepharoplasty incisions; however, the scarring problem cannot be resolved. The lateral extension of the lower transconjunctival incision with lateral canthotomy has the potential for complications, such as lateral canthal malposition, entropion, ectropion, and visible scarring.

Fixation at the lateral orbital rim was not considered mandatory in all tripod ZMC fractures. In our surgical strategy, additional fixation of the frontozygomatic region was selectively performed when a palpable step deformity or instability persisted at the lateral orbital rim despite adequate reduction and fixation through the gingivobuccal sulcus approach. In this clinical setting, the upper eyelid transconjunctival approach proved to be a useful surgical option, allowing secure fixation of the frontozygomatic suture without the need for an additional cutaneous incision.

To minimize these potential drawbacks, we propose an upper eyelid transconjunctival incision as an alternative to the lateral brow incision for frontozygomatic suture fixation in two-point fixation. The upper eyelid transconjunctival approach provides a sufficient surgical field and working space for manipulation of the zygomaticofrontal buttress. Concerns may arise regarding potential injury to the levator aponeurosis and lacrimal gland; however, careful preseptal dissection, similar to that used in the lower eyelid transconjunctival approach, minimizes the risk of exposure of these structures. In addition, the non-scar two-point fixation technique does not substantially prolong operative time, requiring approximately 15 to 20 minutes, while potentially avoiding visible external scarring.

Our postoperative results also demonstrated acceptable data compared with traditional lateral eyebrow incisions regarding bony and soft tissue intermalar height, without significant differences after 6 months. In other words, the incision in the non-scar fixation technique on the upper lateral conjunctiva provides a sufficient operative field for the reduction of the fractured ZMC and fixation of the miniplate or microplate in an accurate position without external evidence of repair. A 1.5-cm incision at the upper lateral conjunctiva was sufficient for the reduction of the zygomaticofrontal buttress and insertion of a 4-hole 1.5-mm miniplate.

In the present study, no clinically significant ophthalmic complications were observed during the follow-up period. Specifically, no cases of ptosis, impairment of eyelid closure, tear dysfunction, conjunctival scarring, or lateral canthal and soft tissue asymmetry were identified. Potential concerns with the upper eyelid transconjunctival approach include injury to the levator apparatus, postoperative eyelid dysfunction, and conjunctival complications. However, in our technique, the incision is placed at the lateral margin of the upper tarsal plate, superior to the lateral canthal tendon, allowing access to the zygomaticofrontal buttress through a relatively limited and targeted operative field. This anatomical pathway may help minimize unnecessary manipulation of adjacent periocular structures. Minor conjunctival findings, including chemosis and subconjunctival hemorrhage, were observed in a small number of patients, but all resolved spontaneously within a few days without intervention. These findings suggest that the approach can be performed safely when careful dissection and appropriate patient selection are maintained.

Patients in the control group underwent fixation through preexisting periorbital traumatic lacerations, and therefore, the postoperative scars represented sequelae of the initial trauma rather than surgically induced scars. As such, the size and appearance of these scars varied depending on the injury mechanism and wound condition, which limits direct comparison with a scarless surgical approach such as the upper eyelid transconjunctival incision. Accordingly, the control group in this study was not intended for direct comparison of scar outcomes, but rather to evaluate whether adequate anatomical reduction and stable fixation could be achieved using the upper eyelid transconjunctival approach. This also represents a major limitation of the present study, as the absence of a true control group treated with a conventional transcutaneous approach inherently limits direct comparison of access-related advantages, including surgical exposure and aesthetic outcomes.

Because comminuted or highly complex ZMC fractures were excluded, the applicability of this approach to such fracture patterns could not be assessed. In these cases, wider exposure and more extensive fixation may be necessary. Further studies are needed to determine whether this technique can be safely extended to more complex fractures.

Another limitation of this study is that the follow-up period of 6 months may be insufficient to evaluate long-term stability, particularly in cases where absorbable fixation materials are used. As this study is based on a retrospective review, the duration of follow-up was inherently limited. Further studies with longer follow-up periods are needed to confirm the long-term safety and durability of this technique.

Despite these limitations, the non-scar fixation technique using an upper lid transconjunctival incision appears to be a reliable and safe surgical option for selected ZMC tripod fractures.
